# Variations of the origin of collateral branches emerging from the posterior aspect of the brachial plexus

**DOI:** 10.1186/1749-7221-2-14

**Published:** 2007-06-23

**Authors:** Luis Ernesto Ballesteros, Luis Miguel Ramirez

**Affiliations:** 1 Medicine Faculty, Universidad Industrial de Santander, Bucaramanga, Colombia

## Abstract

**Background:**

The frequency of variation found in the arrangement and distribution of the branches in the brachial plexus, make this anatomical region extremely complicated. The medical concerns involved with these variations include anesthetic blocks, surgical approaches, interpreting tumor or traumatic nervous compressions having unexplained clinical symptoms (sensory loss, pain, wakefulness and paresis), and the possibility of these structures becoming compromised. The clinical importance of these variations is discussed in the light of their differential origins.

**Methods:**

The anatomy of brachial plexus structures from 46 male and 11 female cadaverous specimens were studied. The 40–80 year-old specimens were obtained from the Universidad Industrial de Santander's Medical Faculty's Anatomy Department (dissection laboratory). Parametric measures were used for calculating results.

**Results:**

Almost half (47.1%) of the evaluated plexuses had collateral variations. Subscapular nerves were the most varied structure, including the presence of a novel accessory nerve. Long thoracic nerve variations were present, as were the absence of C5 or C7 involvement, and late C7 union with C5–C6.

**Conclusion:**

Further studies are needed to confirm the existence of these variations in a larger sample of cadaver specimens.

## Background

Brachial plexus (BP) anatomical variations have been described in humans by many authors, although such variations have not been extensively catalogued [1-4]. Variations in plexus patterns may be due to unusual formation during the development of trunks, divisions, or cords [5]. The more common BP variations occur at the junction or separation of the individual parts [6,7]. Peripheral (collateral) nerves arise from the whole plexus trajectory. These collaterals reach proximal regions exclusively innervating some scapular belt muscles.

Anesthetic blocks, surgical approaches, the interpretation of a nervous compression having unexplained clinical symptoms (sensory loss, pain, wakefulness and paresis), and these structures being compromised represent the clinical importance of these variations [8,9]. Dorsal scapular, long thoracic, suprascapular, subscapularis and thoracodorsal origins were studied to show variable BP collateral arrangements.

## Methods

BP structures from 46 male and 11 female mixed-race, cadaverous specimens (40–80 years old) were studied. These specimens were obtained from the Medical Faculty's Department of Anatomy (dissection laboratory) at Universidad Industrial de Santander during academic semesters in 2005 and 2006. The cause of death for each cadaver was not known in detail. The specimens enter the dissection laboratory as donated material which relatives have not claimed from the University Hospital. None of them had suffered pathological lesions, traumatic lesions or surgical procedures in the neck, thoracic or axillary region. All specimens were fixed in 10% formaldehyde solution. The dissections were performed by the authors involving three specific areas: neck, antero-lateral thorax and delto-scapular area. The origin of the BP collaterals were carefully dissected from the cervico-thoracic roots to the final muscular innervation contact. Dorsal scapular, long thoracic, suprascapular, subscapularis and thoracodorsal nerves were dissected separately at the end of the procedure. Pectoralis medial and lateral branches, and the subclavian nerve were not included due to difficulty of accessing the posterior aspect of the BP. This included the first plane of dissection that involved pectoralis major and minor muscles and the clavicle with its respective innervation. Each finding was ordered, photographed, and registered according to gender, side, level of union and collateral origin, and the presence of additional collaterals. The results were descriptive, and data was presented as absolute numbers and percentages. The origin of each studied nerve was categorized as having a usual origin (illustrated in classical texts) or a variation (Table 1, Table 2).

**Table 1:** Frequency of subscapular nerves origins.

*Upper subscapular*	*n (%)*
From posterior cord (usual)	19 (33.9)
From axillary	3(5.4)
From PD of ST	28 (50)
From ST and MT	2 (3.6)
From others: suprascapular or C8	4 (7.1)
***Lower subscapular***	***n (%)***
From posterior cord (usual)	18 (31.6)
From axillary	31 (54.4)
From radial	1 (1.7)
From thoracodorsal	7 (12.3)
***Thoracodorsal***	***n (%)***
From posterior cord (usual)	44 (78.6)
From radial	5 (8.9)
From MT	2 (3.6)
From axillary	5 (8.9)
***Accessory subscapular***	***n (%)***
From posterior cord	12 (21.1)
From ST	6 (10.6)
From axillary	4 (7)
Absent	35 (61.3)

**Table 2:** Frequency of origins of long thoracic, suprascapular and dorsal scapular nerves.

*Long thoracic*	*n (%)*
From C5, C6, C7 -above the 1^st ^rib (early union) – (usual)	11 (20.4)
From C5, C6, C7 -above the 3^rd ^rib (late union)	6 (11.1)
C5 (dorsal scapular common trunk), C6, C7-(early union)	7 (13.0)
C5 (dorsal scapular common trunk), C6, C7-(late union)	12 (22.2)
C5 not supplying	6 (11.1)
C7 not supplying	10 (18.5)
without union (C5, C6 + C7/C5 + C6, C7)	2 (3.7)
***Suprascapular***	***n (%)***
From ST (usual)	47 (82.4)
From C5	9 (15.8)
From C5 and ST	1 (1.8)
***Dorsal scapular***	***n (%)***
From unique ramus (usual)	10 (17.9)
From common trunk with long thoracic	17 (30.4)
From C4	16 (28.6)
From C4, C5	13 (23.1)

## Results

**Figure 1:**
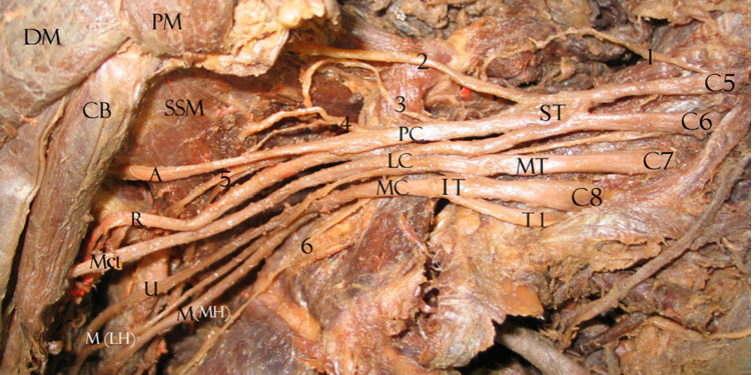
Right BP with collateral origin variations for upper subscapular nerve and an accessory subscapular nerve. C5, C6, C7, C8, T1: spinal nerve ventral rami. ST: superior trunk. MT: middle trunk. IT: inferior trunk. LC: lateral cord. PC: posterior cord. MC: medial cord. A: axillary nerve. R: radial nerve. Mct: musculocutaneous nerve. U: ulnar nerve. M(LH): lateral head of median nerve. M(MH): medial head of median nerve. PM: pectoralis minor muscle. SSM: subscapularis muscle. DM: deltoid muscle. 1: dorsal scapular nerve.2: suprascapular nerve. 3: upper subscapular nerve (originating from the ST posterior cord). 4: accessory subscapular nerve. 5: lower subscapular nerve. 6: long thoracic nerve.

**Figure 2:**
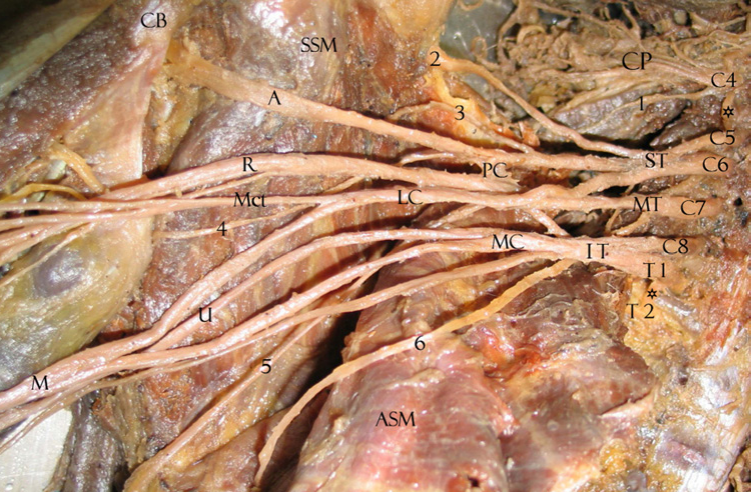
Right BP with collateral origin variations for lower subscapular nerve and thoracodorsal nerve. C4, C5, C6, C7, C8, T1, T2: spinal nerve ventral rami with prefixed C4 (asterisk) and postfixed T2 (asterisk). ST: superior trunk. MT: middle trunk. IT: inferior trunk. LC: lateral cord. PC: posterior cord. MC: medial cord. A: axillary nerve. R: radial nerve. Mct: musculocutaneous nerve. U: ulnar nerve. M: median nerve. ASM: serratus anterior muscle. SSM: subscapularis muscle. CP: cervical plexus. 1: dorsal scapular nerve (originating from C4). 2: suprascapular nerve. 3: upper subscapular nerve. 4: lower subscapular nerve (originating from the axillary nerve). 5: thoracodorsal nerve originating from MT posterior division. 6: long thoracic nerve.

**Figure 3:**
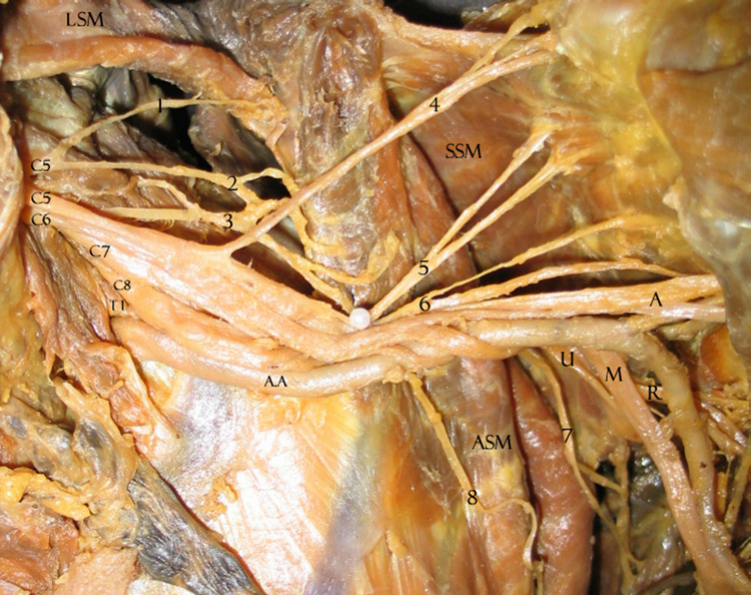
Left BP with collateral origin variations for shared C5. C5, C6, C7, C8, T1: spinal nerve ventral rami. AA: axillary artery. LSM: levator scapula muscle. SSM: subscapular muscle. ASM: anterior serratus muscle. A: axillary nerve. R: radial nerve. U: ulnar nerve. M: median nerve.1: dorsal scapular nerve. 2: ramus from C5 to long thoracic (sharing the same root with dorsal scapular). 3: C6 ramus to long thoracic. 4: suprascapular nerve. 5: upper subscapular nerve. 6: lower subscapular nerve. 7: thoracodorsal nerve. 8: long thoracic nerve.

**Figure 4:**
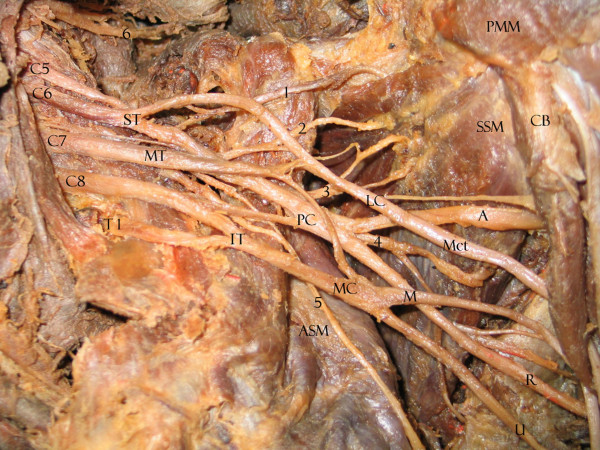
Left BP with collateral origin variations for subscapular nerves. C5, C6, C7, C8, T1: spinal nerve ventral rami. ST: superior trunk. MT: middle trunk. IT: inferior trunk. LC: lateral cord. PC: posterior cord. MC: medial cord. A: axillary nerve. R: radial nerve. Mct: musculocutaneous nerve. U: ulnar nerve. M: median nerve. ASM: serratus anterior muscle. SSM: subscapularis muscle. PMM: pectoralis minor muscle. 1. suprascapular nerve. 2: upper subscapular nerve (originating from the ST posterior cord). 3: accessory subscapular nerve. 4. lower subscapular nerve (originating from the thoracodorsal nerve). 5: long thoracic nerve. 6: dorsal scapular nerve (originating from C4).

We found that 33.9% of upper subscapular nerves, 31.6% of lower subscapular nerves, 78.6% of thoracodorsal nerves, 20.4% of long thoracic nerves, 82.4% of suprascapularnerves and 17.9% of dorsal scapular nerves had the usual origins expected for BP collaterals. Tables 1, 2 and 3 show the variations, summarizing all collateral origin variations in an ordered form. Variations were not bilateral in any cadaver. The most variable structures are seen in Figures 1, 2, 3, 4. Briefly, 54.4% of the lower subscapular nerves originated from the axillary nerve and 12.3% from the thoracodorsal nerve (Fig 1). 50% of the upper subscapular nerves originated from the posterior division (PD) of the superior trunk (ST) (Fig 2), 5.4% from the axillary nerve, and 3.6% from the union of the ST and middle trunk (MT) (Table 1). Other variations included a common C5 nerve ramus forming the long thoracic and dorsal scapular nerves (35.2%) (Table 2; Fig 3), a late union below the first rib from C7 with C5–C6 forming the long thoracic nerve (33.3%), a suprascapular nerve originating from the C5 ventral ramus (15.8%), a thoracodorsal nerve originating from the radial nerve (8.9%), an accessory subscapular nerve in 38.7% of the samples (Fig 4), and the accessory subscapular nerve was observed coming from the posterior cord in 21.1% of the samples.

**Table 3:** Percentage of BP collateral origins (usual and variable presentation).

BP collateral	Usual n (%)	Variation n (%)
Long thoracic	18 (33.3)	36 (66.6)
Suprascapular	47 (82.5)	10 (17.5)
Dorsal scapular	27 (48.2)	29 (51.8)
Upper subscapular	19 (33.9)	37 (66.1)
Thoracodorsal	44 (78.6)	12 (21.4)
Inferior subscapular	18 (31.6)	39 (68.4)
Accessory subscapular	35 (61.4)*	22 (38.6)

*Absent collateral (usual)

There were no significant differences regarding gender or side where the variations were found (p = 0.876 and 0.523, respectively).

## Discussion

BP variations regarding collateral branches are common, but have not been fully reported in several investigations. These results reinforce the concept that anatomical variations are so common that normal textbook parameters must be treated carefully, especially regarding surgical procedures. Uzun *et al*. [10] have stated that BP variations could be prevented during extensive surgical procedures in the neck and axilla which are considered vulnerable areas involving legal repercussions [11,12]. Uysal *et al*. [13] found 53.5% of human fetuses have different variations in the BP. Our results were similar in that 47.1% of the evaluated plexuses had collateral variations. Regarding side and gender, controversial results have been returned due to Uysal *et al*. [13] and Kerr [14] affirming that variations were more likely in females and on the right-hand side. However, Uzun *et al*. [10] and Fazan *et al*. [15] found no differences. Our results revealed no discrepancy concerning this variable.

A classical description of the long thoracic nerve (LTN) deals with a nerve formed by an upper portion originating from C5 and C6 nerve roots and a lower portion coming from C7. The union of the upper and lower portions are normally linked to either the axilla (an extensive, not well-discriminated area) or the upper border of the anterior serratus muscle (first rib). Tubbs *et al*. [16] found that 61% of cadaverous specimens had C5, C6 and C7 union at the second rib posterior to the axillary artery. They also found this union was at the first rib level posterior to the MT of the BP in 33% of cases. In addition, they also found that the C5 element of the LTN did not join with C6 and C7 and traveled directly to the serratus anterior muscle in 6% of cases. We found this same component in 3.7% of our samples.

Several surgical procedures including first rib resection, lung surgery, transaxillary thoracotomy, and chest tube placement might cause injury to the long thoracic nerve [4,17]. Our results showed a significant late union in 18 cases (33.3%). This might explain partial lesions in a patient's clinical symptoms when these nerves are injured, resulting in such morphological expression. A detailed picture of upper and lower portion union (late or proximal) must thus be provided to avoid damage by a surgeon who counts on such description. Familiarity with a variation's origin and the trajectory of collateral nerves emerging from the posterior aspect of the BP (i.e. subscapularis and thoracodorsal) must be taken into account by a surgeon during procedures such as radical mastectomy with axillary emptying to prevent unfavorable iatrogenic outcomes. Besides the presence of an accessory subscapular nerve, understanding the late or lateral origin of a collateral nerve emerging from radial or axillary nervesis vital for evading positive motor signs, such as compromising arm adduction, extension or medial rotation.

Moreover, C5 (11%) and C7 (18%) do not always contribute to the long thoracic nerve. Lee *et al*. [18] found that C5 (11.3%) and C7 (7.7%) were not necessarily contributing components. Horwitz and Tocantins [19] and Hovelacque [20] found the C7 component of the LTN absent in 8% and 1.7% of cases, respectively. Although other studies found additional contributions from C4 and C8 to the LTN in a small number of cases, we could not identify them in this study [18,19,23].

Our results regarding the dorsal scapular nerve fit the classical description of originating from C5 in only 48.3% of the samples. We also found an origin from a common trunk with the LTN (30.4%) in fewer cases than Horwitz and Tocantis [19] (44%). Interestingly, Lee *et al*. [18] described a classical dorsal scapular nerve in 75.8% of the samples, as well as finding a variation spectrum consisting of 9% originating from ST, 7.6% from C4, C5 and 7.6% from C6. Tubbs *et al*. found dorsal scapular nerve originating from C5 in 95% of cases and the rest from C5, C6 spinal nerves [21]. Mallesy *et al*. confirmed the additional cervical plexus supply (C3, C4) to the levator scapula muscle, just as anatomical textbooks state [22]. Interestingly, Yan *et al*. stress the real and apparent origins of spinal BP segments, detailed work showing an additional source of upper spinal segment fibers to the BP and their anterior and posterior arrangement refuting macroscopic BP arrangement [23].

Regarding the suprascapular nerve, we found little variation from the classical description (82.4%). Lee *et al*. [18] found it originating from C5 in one case, and Fazan *et al*. [14] found the same origin in 5.5% of cases. Lee *et al*. [18] also found the suprascapular nerve originating from C6 (1.3%) and C4 contributing to C5 (7.2%). Yan *et al*. showed that anterior and posterior contributions form the spinal segment of the BP to the suprascapular nerve [24]. We believe that the most rostral motor contribution (C4) to this nerve might be consistent with a shared lesion when this medullar segment is involved with a biomechanical effect on shoulder mobility and diaphragmatic function (phrenic nerve).

The thoracodorsal nerve originated from the posterior cord (PC) in 78.6% of samples in the present study. A classical description of the thoracodorsal nerve origins involves three different origins including the MT (3.6%), radial (8.9%), and axillary (8.9%) nerves. Fazan *et al*. [15] found an axillary origin or a radial nerve origin in 13% and 5.5% of cases, respectively. Tubbs *et al*. found 1.5% of thoracodorsal nerves coming from the radial nerve [25]. Trauma of the posterior wall of the axillary region could harm latisimus dorsis muscle function (humeral movement extension, adduction and medial rotation), depending on lesion level and the involvement of its several origins. For instance, an axillary nerve lesion engaging the thoracodorsal nerve origin may produce a more extensive functional lesion including latisimus dorsi, deltoid and teres minor muscles.

The upper subscapular nerve presented broad variability in our findings. The most frequent result did not fit the classical description of a PC origin in 50% of the cases. We found 33.9% originating from the PC and the rest from axillary, suprascapular and C8 spinal nerves. Lee *et al*. [18] considered upper subscapular nerves originating from the axillary nerve to be very infrequent. However, Fazan *et al*. [15] observed an axillary origin in 5.5% of cases and Tubbs *et al*. [26] found it in 3%, which was similar to our results.

Even though Tubbs *et al*. [26] reported that the lower subscapular nerve originates directly from the axillary nerve's proximal segment, we found this late origin in only 54.4% of samples. This is in agreement with Fazan *et al*. who found this at a rate of 54% [15]. Although Lee *et al*. [18] only observed occasional lower subscapular nerve sources coming from the thoracodorsal nerve, we found 12% of this configuration in our study and Fazan *et al*. observed 7% as well [15].

Among several findings in some of the BP collaterals, the present study confirms the existence of the accessory subscapular nerve. Even though this nerve has been noted in previous studies, none of them have classified it or calculated its incidence (38.7%), originating mostly from the posterior cord (21.1%).

There must be full awareness of the origin of variation in collateral branches from the posterior aspect of the BP and their configuration due to their significance in interpreting diagnostic images, nerve blocks, traumatic damage and surgical approaches. Unexplained clinical symptoms, such as sensory loss, pain, wakefulness and paresis compromising these structures, challenge diagnosis. Descriptions of such nerve variations are thus pertinent for anatomists, anesthesiologists, radiologists and surgeons.

## Conclusion

The anatomical basis of BP collateral variations should be kept in mind, especially while performing surgical exploration, especially in the axillary and neck region and when interpreting clinical symptoms following trauma or tumor events. Being aware of the restrictiveness of this study's sample size, we have concluded from this study that:

Almost half the evaluated plexus had collateral variations (47.1%);

Subscapular nerves were the most varied structure, including the presence of an accessory nerve;

Long thoracic nerve variations were present as were the absence of C5 or C7 involvement and late C7 union with C5–C6;

Further studies are needed to confirm the existence of these variations in a larger sample of cadaver specimens.

## Abbreviations

Brachial plexus (BP)

Superior trunk (ST)

Middle trunk (MT)

Inferior trunk (IT)

Anterior divisions (AD)

Posterior divisions (PD)
